# Functional characterization of Kv11.1 (hERG) potassium channels split in the voltage-sensing domain

**DOI:** 10.1007/s00424-018-2135-y

**Published:** 2018-03-23

**Authors:** Pilar de la Peña, Pedro Domínguez, Francisco Barros

**Affiliations:** Departamento de Bioquímica y Biología Molecular, Universidad de Oviedo, Edificio Santiago Gascón, Campus de El Cristo, 33006 Oviedo, Asturias Spain

**Keywords:** Potassium channel, Split channel, Gating, hERG, Voltage sensor

## Abstract

Voltage-dependent KCNH family potassium channel functionality can be reconstructed using non-covalently linked voltage-sensing domain (VSD) and pore modules (split channels). However, the necessity of a covalent continuity for channel function has not been evaluated at other points within the two functionally independent channel modules. We find here that by cutting Kv11.1 (hERG, *KCNH2*) channels at the different loops linking the transmembrane spans of the channel core, not only channels split at the S4–S5 linker level, but also those split at the intracellular S2–S3 and the extracellular S3–S4 loops, yield fully functional channel proteins. Our data indicate that albeit less markedly, channels split after residue 482 in the S2–S3 linker resemble the uncoupled gating phenotype of those split at the C-terminal end of the VSD S4 transmembrane segment. Channels split after residues 514 and 518 in the S3–S4 linker show gating characteristics similar to those of the continuous wild-type channel. However, breaking the covalent link at this level strongly accelerates the voltage-dependent accessibility of a membrane impermeable methanethiosulfonate reagent to an engineered cysteine at the N-terminal region of the S4 transmembrane helix. Thus, besides that of the S4–S5 linker, structural integrity of the intracellular S2–S3 linker seems to constitute an important factor for proper transduction of VSD rearrangements to opening and closing the cytoplasmic gate. Furthermore, our data suggest that the short and probably rigid characteristics of the extracellular S3–S4 linker are not an essential component of the Kv11.1 voltage sensing machinery.

**Electronic supplementary material:**

The online version of this article (10.1007/s00424-018-2135-y) contains supplementary material, which is available to authorized users.

## Introduction

Kv11.1 (hERG, *KCNH2*) K^+^ channels mediate the cardiac *I*_Kr_ current that acts as an important determinant of action potential repolarization in the human ventricle and of pacemaking activity in heart nodes [17, 34, 45, 58]. Impairment of Kv11.1 function by mutations in the *KCNH2* gene or by a variety of drugs prolongs the QT interval of electrocardiograms leading to inherited and acquired type 2 long QT syndrome, increasing the risk of torsade de pointes arrhythmia, ventricular fibrillation and sudden cardiac death [46, 58]. Furthermore, Kv11.1 is also expressed in a variety of non-cardiac cells in which it plays a key role in setting their electrical behaviour [4, 5, 40, 58].

Kv11.1 belongs to the voltage-gated family of potassium channels (Kv), all of them characterized by a tetrameric molecular architecture in which each subunit contains six transmembrane helices (S1–S6) with a modular organization [7, 52, 68]. In the functional complete channel, the pore domain (PD) is formed by the pore modules (S5–S6 and the intervening pore loop) of all four subunits, which arrange with fourfold symmetry surrounding a unique ion conduction pore. In the assembled tetramer, the PD is also surrounded by four voltage-sensing domains (VSDs) located at the periphery. VSD corresponds to transmembrane helices S1–S4, of which the primary voltage sensitive component is helix S4, containing the positively charged residues that move in response to changes in membrane potential [65]. The concept of Kv channels as a product of a evolutive combination of two functionally autonomous VSD and PD modules [2, 28, 69] is reinforced by (i) the existence of PD-only voltage-independent channels [8, 20, 27, 39] that probably share a common ancestor with Kv and other voltage-dependent channels [23, 29, 69], (ii) the demonstration of functional VSD-only-based voltage-dependent channels [10, 12, 54] and voltage-controlled enzymes in which a VSD resembling those found in voltage-gated channels provides membrane potential control of the catalytic activity [60] and (iii) the generation of voltage-gated ion channels by either fusing together VSDs and PDs from different sources [3, 33, 53] or co-expressing them as separate protein entities [14].

In Kv11.1 and other Kv channels, VSD and PD are covalently linked by the so-called S4–S5 linker, classically viewed as a rigid mechanical lever that transmits the voltage sensor reorganizations triggered by changes in transmembrane voltage to the channel gate located at the bottom of transmembrane helix S6 [9, 59]. However, we have recently shown that voltage-dependent Kv11.1 and Kv101.1 functionality can be reconstructed using non-covalently linked VSD and PD (split channels [50]), pointing to an alternate gating mechanism for this channel diverging from that operating in the more traditionally considered *Shaker*-like Kv channels [14, 32, 55]. Interestingly, in spite of the very short length of the S4–S5 linker in these channels leading to a non-domain swapped architecture unlike that encountered in the ‘more classical’ Kvs 1–9 [65, 67], some differences in functional outputs can be induced when the split point is moved along the linker. Thus, breaks in the S4 helix/S4–S5 linker connection leads to altered VSD-PD coupling inducing constitutively active Kv10.1 splits [55] and strongly hyperpolarization-shifted Kv11.1 splits also showing an increased destabilization of closing, a reduced ability to reach more distal closes state(s) and a reduced voltage dependency of both activation and deactivation [14]. On the other hand, interruptions at the C-terminal section of the S4–S5 linker slow down Kv10.1 activation and deactivation kinetics [55] and give rise to quite normal VSD-PD coupling during activation but markedly accelerated deactivation kinetics in Kv11.1 [14]. Whereas interruptions of the channel protein inside the linker joining the two full-length PD and VSD modules [14, 32, 55], able to act as separate and autonomous functional entities, could be considered a relatively conservative approach, the possibility that a fully functional channel can also be regenerated by combining channel fragments from a protein interrupted at other levels different from the S4–S5 linker has not been evaluated. Therefore, here, we checked this possibility after splitting Kv11.1 at the different loops linking the transmembrane spans of the channel core. Surprisingly, we found that channels covalently interrupted at the intracellular S2–S3 and the extracellular S3–S4 loops, also yield fully functional channel proteins. Careful functional characterization of these constructs suggests that the S2–S3 linker may modulate the transduction of VSD rearrangements to opening and closing of the cytoplasmic channel gate, whereas the covalent continuity of the S3–S4 linker is not essential for proper operation of the Kv11.1 voltage sensing machinery.

## Materials and methods

### Molecular biology, mutagenesis and expression in *Xenopus laevis* oocytes

Kv11.1 split channels were generated as PCR fragments containing the desired coding sequences that were inserted into the pSP64A+ vector as HindIII-BamHI fragments. The N-terminal demi-channel fragment for split 478 was synthesized using a sense oligonucleotide containing a HindIII site, a Kozak signal and the sequences for the initial eight Kv11.1 residues together with the corresponding antisense oligonucleotide carrying the coding sequence for residues 466 to 478, followed by a stop codon and the BamHI recognition site. For the C-terminal demi-channel fragment synthesis, the sense oligonucleotide was designed to contain a HindIII site, a Kozak sequence and the start codon followed by the 479 to 489 Kv11.1 coding sequence, whereas the antisense oligonucleotide covered the last 10 residues of the protein (1049–1059) a stop codon and the BamHI recognition sequence. All the rest of Kv11.1 split channels (438, 482, 514, 518, 573 and 637) were generated in an identical manner but to modify the N-terminal of the constructs the antisense oligonucleotide was designed to contain the desired Kv 11.1 final coding sequence before the stop codon. In the case of the C-terminal constructs, the sense oligonucleotide was designed to contain the corresponding coding sequences of the different C-terminal demi-channels after the start codon.

Split channel constructs with the I521C single-point mutation were created by overlapping PCR as previously described [14–16]. All constructs were analysed by standard fluorescence-based DNA sequencing to confirm the mutations and verify the absence of errors.

Procedures for frog anaesthesia and surgery to obtain oocytes and microinjection have been detailed elsewhere [1, 6, 14–16, 61]. Oocytes were maintained in OR-2 medium (82.5 mM NaCl, 2 mM KCl, 2 mM CaCl_2_, 2 mM MgCl_2_, 1 mM Na_2_HPO_4_, 10 mM HEPES, at pH 7.5). Cytoplasmic microinjections were performed with 50 nl of in vitro synthesized cRNA per oocyte.

### Electrophysiological recording and analysis

Two-electrode voltage clamp recordings were performed as previously described [1, 6, 14–16, 61] in manually defolliculated oocytes at room temperature 2–3 days after injection, using a Turbo TEC-01C amplifier (NPI electronics). The intracellular electrodes had resistances of 0.4–0.8 MΩ when filled with 3 M KCl. Unless otherwise stated, recordings were obtained in OR-2 medium. In some cases, high-K^+^ OR-2 medium in which 50 mM KCl replaced an equivalent amount of NaCl was used to maximize currents of those constructs showing a low level of functional expression. Oocytes showing membrane potentials more positive than − 30 mV after impalement with the first electrode in OR-2 medium were discarded. Current recordings were obtained in an experimental chamber of 0.12 ml volume continuously perfused at 2.4 ml/min. For experiments with [2-(trimethylammonium)ethyl]methanethiosulfonate chloride (MTSET; Biotium) the powder reagent was aliquoted, stored at − 20 °C and dissolved in OR-2 just before application to each individual cell. In this case, silver chloride ground electrodes were connected to the bath chamber through agar bridges. Data acquisition and analysis were performed with the Pulse-PulseFit (HEKA Electronics) and IgorPro (WaveMetrics) software packages running on Macintosh computers. Ionic currents sampled at 1 or 10 kHz were obtained using the voltage protocols indicated in the graphs.

The voltage dependence of activation was assessed by standard tail current analysis using depolarization pulses of variable amplitude. For very rapidly deactivating constructs, fitting the relaxation of the tail currents and extrapolating the magnitude of the decaying current to the time the depolarizing pulse ended were used to determine the amount of current passing through channels opened on depolarization without the influence of rapid inactivation. Tail current magnitudes normalized to maximum were fitted with a Boltzmann function to estimate the *V*_1/2_ and equivalent gating (*z*_*g*_):

$$ {I}_{\mathrm{tail}}/{I}_{\mathrm{max}}=1/\Big[\left(1+\exp \left(\left({V}_{1/2}-V\right){z}_gF/ RT\right)\right] $$

where *V* is the test potential and *F*, *R* and *T* are Faraday constant, gas constant and absolute temperature, respectively. This function was also used to fit the MTSET voltage dependence data.

The time course of voltage-dependent activation was studied using an indirect envelope-of-tail-currents protocol, varying the duration of depolarization prepulses and following the magnitude of the tail currents on repolarization. The time necessary to reach a half-maximal tail current magnitude was used to compare the speed of activation of the different channels.

The rates of deactivation were determined from negative-amplitude biexponential fits to the decaying phase of tail currents using a function:

$$ y={A}_f\exp \left(-\mathrm{inv}{\tau}_f.x\right)+{A}_{\mathrm{s}}\exp \left(-\mathrm{inv}{\tau}_s.x\right)+C $$

where *τ*_*f*_ and *τ*_*s*_ are the time constants of fast and slow components, *A*_*f*_ and *A*_*s*_ are the relative amplitudes of these components and *C* is a constant. In this case, the first cursor of the fitting window was advanced to the end of the initial hook because of the recovery of inactivation.

Onset of fast inactivation was studied after activation and inactivation of the currents with a prepulse to positive voltages, followed by a second short prepulse to around − 100 mV used to recover the channels from inactivation, and a subsequent test pulse at different voltages to re-inactivate the channels. Time constants for the onset of inactivation were obtained from current traces fitting a single-exponential function to the decaying portion of the currents during the test pulses. The voltage dependence of inactivation was determined with an alternative triple pulse protocol in which cells were depolarized to + 40 mV for several seconds to activate/inactivate the channels and subsequently allowed to relax to a inactivation steady state during a brief test pulse at different voltages, followed by a third step to + 40 mV in which the initial current magnitude was measured to assess the relative number of channels available to activate at the end of the test pulse. Due to the fast deactivation at negative voltages during the test pulses, the closing rates were obtained in each oocyte from biexponential fits to the decaying tail currents as indicated above. These rates were used to determine the proportion of channels closed at the end of every test pulse and to correct for closing-induced decreases in the initial current magnitude at the beginning of the third step [58].

For experiments designed to study the accessibility of engineered S4 cysteines to MTSET, we routinely used a brief voltage ramp as a stimulatory step that allowed us to kinetically follow the possible shifts in MTSET-induced voltage dependence, either repetitively pulsing the cells at 5-s intervals or maintaining them without pulsing at the indicated holding potentials. All constructs used for this purpose contained a Cys introduced in position 521 of the upper S4 helix (to follow its modification in the presence of MTSET) and two additional mutations (C445V and C449V) to prevent any inadvertent effect caused by MTSET modification of the endogenous cysteines of the Kv11.1 S1–S2 linker. Due to the differences exhibited by different splits in the magnitude of the shift in voltage dependence or in the extent of closing impairment induced by the MTS reagent, the rates and/or the voltage dependence of modification were subsequently obtained quantifying: (i) the magnitude of the peak current increase during the ramp; (ii) the decrease in the time necessary to reach the current peak during the ramp; (iii) the change in the amount of current recorded at the end of a conditioning voltage step to negative voltages (e.g. − 120 mV), included immediately before or after the ramp; and (iv) the decrease in the ratio of the slopes (rectification factor) obtained from the current traces during the steeper raising phase and the minimum slope phase, both in the middle and at the beginning of the ramps, respectively.

### Statistics

Data values given in the text and in figures with error bars represent the mean ± SEM for the number of indicated cells. Comparisons between data groups were at first performed by parametric Student’s impaired *t* test (two-tailed). When significant differences in standard deviation were present an alternate Welch’s test or non-parametric Wilcoxon or Mann-Whitney test were also used. In all cases, *p* values < 0.05 were considered as indicative of statistical significance.

## Results

### Differential effect of interruptions in different loops linking transmembrane helices on Kv11.1 functional expression

**Fig. 1:**
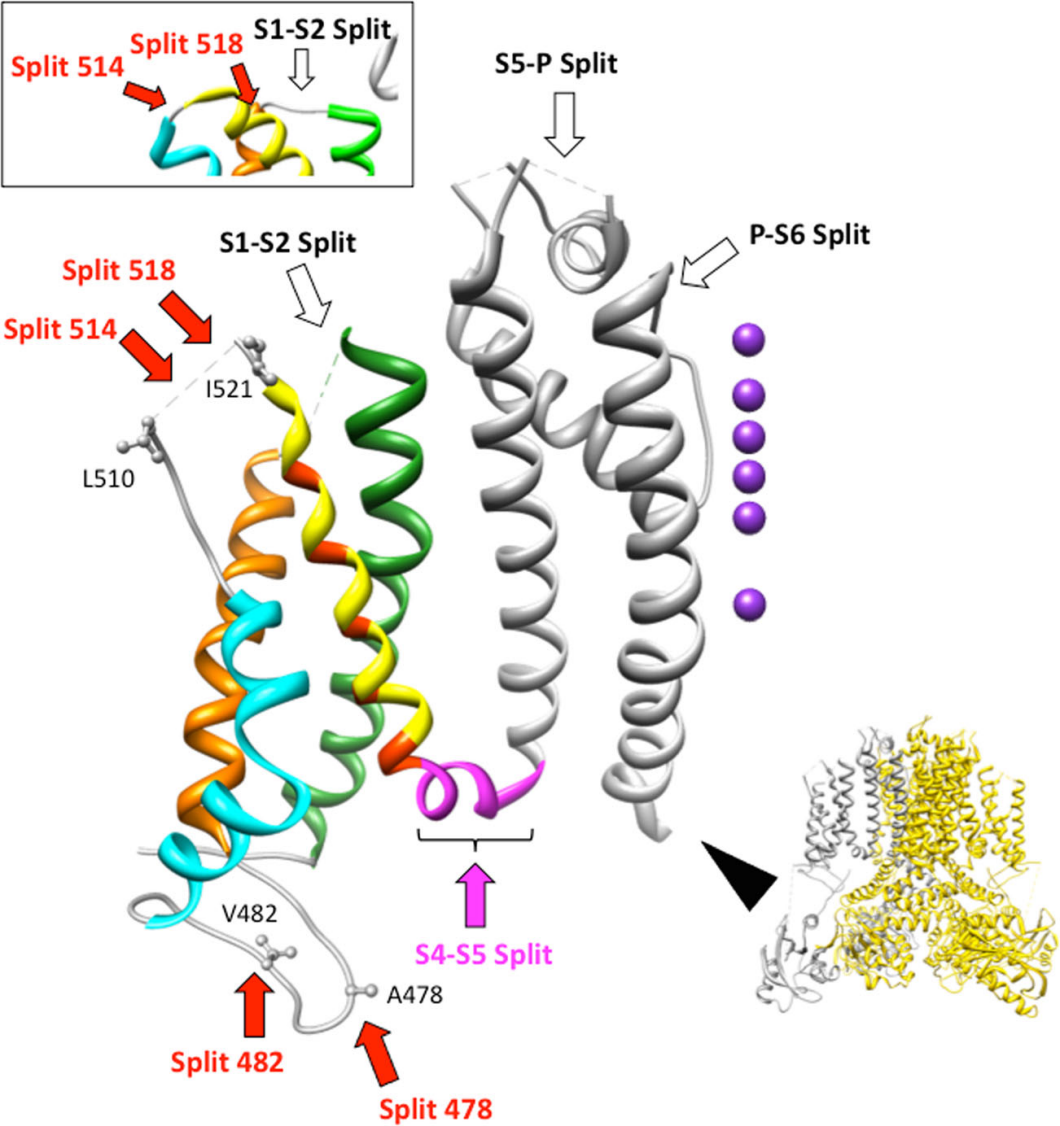
Tridimensional structure of a Kv11.1 subunit transmembrane core region and position of the split points at the different extra and intracellular loops linking the transmembrane segments. A lateral view of the Kv11.1 tetrameric structure ([65]; PDB code 5VA2) with the subunit corresponding to the enhanced view in the main panel highlighted in gray, is shown on the lower right. The region coloured in gray in the central panel corresponds to the pore domain including the S5 and S6 helices. The S4–S45 linker is in magenta. Ribbons from the S4, S3, S2 and S1 segments are in yellow, cyan, brown and green, respectively. The position of the residues providing the positive charges to the S4 helix is marked in orange. Lateral chains of residues 478 and 482 at which the S2–S3 linker was sectioned in this study, and 510 and 521 that mark the boundaries of the structurally undefined S3–S4 linker are shown as ball and stick. The location of the different split points described in this study are indicated, with those that did not yield functional expression marked with open arrows and those giving rise to functional channels marked with solid arrows. A view of the extracellular and structurally defined S1–S2 and S3–S4 linkers region from the homologous Kv10.1 channel ([67]; PDB code 5K7L) is depicted in the upper inset, showing the corresponding position of the Kv11.1514 and 518 splits. Structures were processed with UCSF Chimera [41]

Fully functional Kv11.1 channels are generated when non-covalently linked VSD and PD modules (split channels) are expressed in *Xenopus* oocytes, albeit showing some kinetic differences as a function of the situation of the breaking point along the S4–S5 linker [14, 32]. To check if functionality is only provided by covalent breaks in the linker joining the two modules (known to constitute separate and autonomous functional units), and to gain some insights about the molecular requirements for correct assembly, trafficking and/or insertion of the two channel halves in the membrane, we studied the effects of changing the split point location to other intra and extracellular loops linking the transmembrane helices of the channel core (Fig. 1).

Kv11.1 splits interrupted at the extracellular S1–S2 linker (split 438) and at the external loops linking either the S5 helix with the intervening pore loop that surrounds the selectivity filter (split 573), or the pore loop and the N-terminal end of helix S6 (split 637), were not functionally expressed. Surprisingly, robust voltage-dependent Kv11.1-like potassium currents were observed upon co-injection of cRNAs encoding two demi-channels leading to an N-terminal half truncated in the intracellular S2–S3 linker and a C-terminal half covering the rest of the protein up to the final residue 1159 (S2–S3 linker splits) giving rise to fully functional Kv11.1 channel expression. Also, a combination of the N-terminal part of the channel broken at the extracellular S3–S4 linker together with a second half consisting of the remainder of the S3–S4 linker through the C-terminus (S3–S4 linker splits) yielded functional channels. To ensure that the results are not exclusively due to the unique behaviour of a split made at a particular position, in both cases, two different splits were generated in each linker. For the S2–S3 linker, these corresponded to split positions 478 and 482 (Fig. 1). Since the exact positioning and tridimensional appearance of the S3–S4 linker has not been defined in the recently reported cryo-EM structure of Kv11.1 [65], we also characterized two splits in this loop: split 514 interrupted after the position that marks the middle of the very short S3–S4 linker in the highly homologous Kv10.1 channel ([67], see upper inset of Fig. 1), and split 518 in which the breaking point is located few residues upstream of position 521 in the Kv11.1 S4 helix that becomes extracellularly extruded from the lipid bilayer when the membrane is depolarized [14, 18]. As previously noticed with Kv11.1 and Kv10.1 channels split at the S4–S5 linker [13, 32, 55], no detectable currents were observed when any of the demi-N or demi-C channel messages alone were injected without their complementary demi-channel counterpart. Indeed, a total absence of functional expression was also obtained when the C-terminal halves of the splits 482 in the S2–S3 linker and 518 in the S3–S4 linker carrying a S620T single mutation in the pore (previously shown to ablate inactivation and substantially enhance Kv11.1 channel expression [26, 61]) were separately injected in the oocyte. This indicates that, providing that the C-terminal half of the channel is expressed on the oocyte surface when injected alone as directly demonstrated with the demi-C portion of Kv10.1 split at the S4–S5 [32, 55], an isolated pore domain is not able to work as a voltage-dependent channel if it only carries a partial section (e.g. the S3 and S4 helices) of the voltage sensor. It also suggests that apposition of complete voltage sensor and pore modules is required to form a complex able to yield a functional, voltage-dependent channel.

### Effect of S2–S3 linker interruptions on functional characteristics of the Kv11.1 split channels

**Fig. 2:**
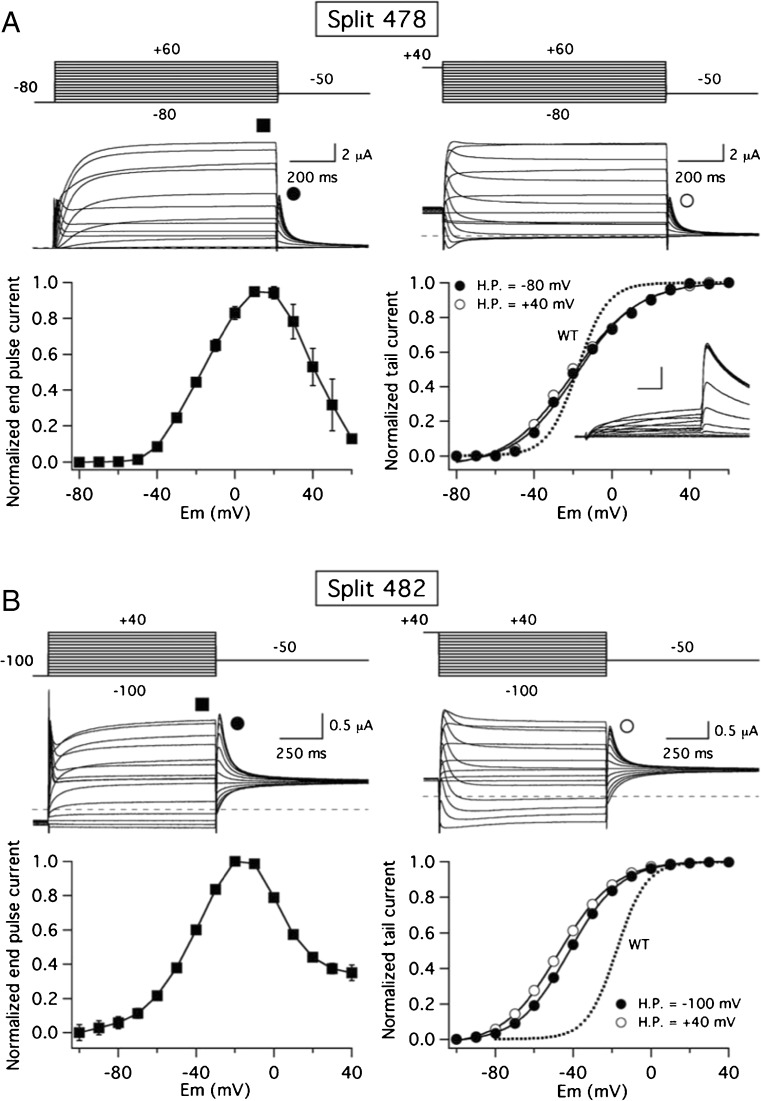
Characterization of activation gating voltage dependence of S2–S3 linker splits. **a** Split 478. Representative membrane currents from individual oocytes submitted to 1 s depolarization pulses to different potentials at 10 mV intervals from a negative (− 80 mV, left) and a positive (+ 40 mV, right), followed by a repolarization step to − 50 mV. Currents recorded without leak subtraction are shown. Averaged *I* vs *V* relationships measured at the end of the depolarization step (signalled by the solid square at the top) and at the peak of the tail current (*G*/*V* plots, circles) are shown at the bottom. Continuous lines in the *G*/*V* plots are Boltzmann fits to the data as indicated in ‘Materials and methods’. A *G*/*V* plot from continuous wild-type channels (WT, dashed line) is also shown for comparison. A family of wild-type channel currents in response to 1-s depolarizing pulses from − 80 to + 40 mV, followed by a repolarization to − 70 mV, is shown in the inset. In this case, the horizontal and vertical scale bars correspond to 200 ms and 500 nA, respectively. **b** Split 482. Recording of currents in response to the protocols shown on top of the traces and analysis were performed as detailed in A

To study the voltage dependence of current activation, we applied 1-s depolarizing pulses to different voltages from a negative holding potential (− 80/− 100 mV), followed by a hyperpolarization step to negative potentials, and plots of normalized currents measured both at the end of the depolarizing step (I/V plot) and at the peak of the tail elicited during the hyperpolarizing pulse (G/V plot) were generated. As shown in Fig. 2a, the potential for half-maximal isochronal activation of the split 478 (*V*_1/2_ of the *G*/*V* plot − 18.3 ± 0.7 mV, *n* = 8) was very similar to that exhibited by the wild-type continuous channel (− 22.2 ± 0.9 mV, *n* = 6). However, significantly left-shifted plots were obtained (*V*_1/2_ = −37.0 ± 0.4 mV, *n* = 15) when the split point was displaced to position 482 in the S2–S3 linker (Fig. 2b). Interestingly, in both cases, a smaller amount of equivalent gating charges (*z*_*g*_ values) estimated from the slope of the *G*/*V* curves was observed (1.78 ± 0.05 and 1.89 ± 0.03 for the 478 and 482 splits, respectively) as compared with those of the WT channel (2.5 ± 0.03). The negatively shifted conductance-to-voltage plot exhibited by the split 482 and the decreased *z*_*g*_ values are reminiscent of the activation gating alterations observed with splits interrupted at the S4 helix/S4–S5 linker interface (e.g. splits 540 and 539, see [14]). These last constructs are also characterized by a complete absence of the so-called mode-shift behaviour, in which the voltage dependencies of current activation and deactivation are clearly separated, as demonstrated by the strong separation of the *G*/*V* plots when they are generated from hyperpolarizing (e.g. − 80/− 100 mV) and depolarizing (e.g. + 40 mV) holding potentials [14]. Therefore, we also compared the position of the *G*/*V* plots after submitting the 478 and 482 split channels to 1-s depolarization pulses to + 40 mV in 10 mV increments, not only from hyperpolarizing, but also from depolarizing (+ 40 mV) holding potentials, followed by the repolarizing voltage step to negative potentials to quantify the tail current magnitudes. As shown in Fig. 2, in both cases, no shift of *G*/*V V*_1/2_ values is observed, indicating that the interruptions at the S2–S3 linker also cause an abolishment of the mode-shift behaviour.

**Fig. 3:**
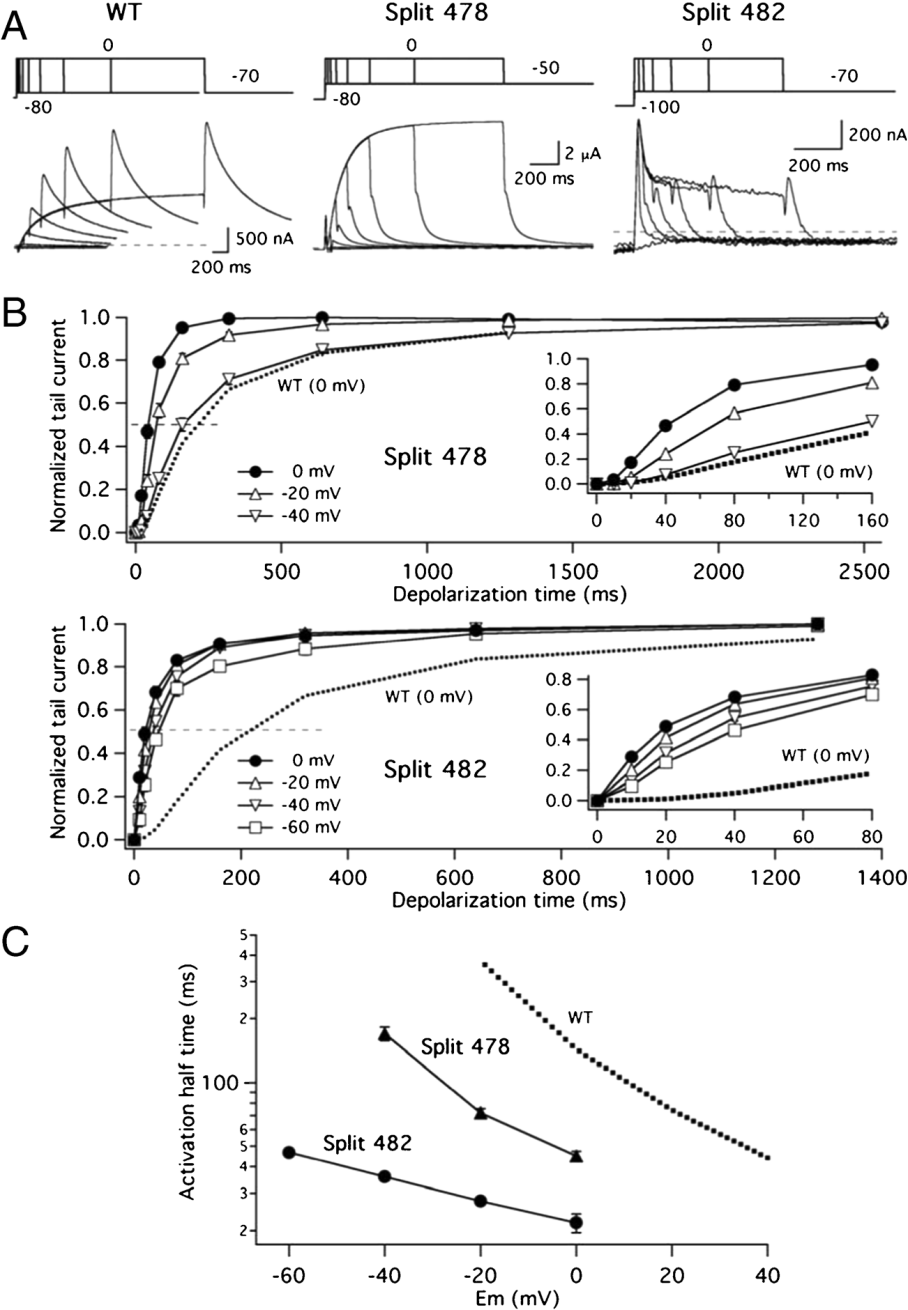
Characterization of voltage-dependent activation rates of S2–S3 linker splits. **a** Time course of current activation at 0 mV. Families of representative membrane currents from individual oocytes are shown. The duration of a depolarizing prepulse to 0 mV was varied as illustrated in the envelope-of-tail-currents protocol at the top, followed by a repolarization step to the indicated potentials. Cells expressing wild-type and split 478 channels were held at − 80 mV and those expressing split 482 channels at − 100 mV to ensure that they always started in a deactivated state. **b** Averaged plots of normalized tail current magnitudes vs depolarization time at different potentials. Most error bars are smaller than the symbols. Values from continuous wild-type channels at 0 mV are shown as a dotted line for comparison. Expansions of the initial tens of ms to highlight the early current delay in the sigmoidal activation time courses are shown in the insets. **c** Comparison of activation rate voltage-dependence. Plots of normalized tail current magnitudes vs depolarization times (**a**) were used to measure times necessary to attain half-maximum current magnitudes, that are plotted vs depolarization potential. Values from continuous wild-type channels (WT) are shown as a dotted line for comparison

Recent data from our laboratory with Kv11.1 splits interrupted at different positions within the S4–S5 linker demonstrated that the characteristic initial delay that precedes the exponential activation time course of the wild-type currents is progressively reduced when the split point is moved towards the beginning of the linker, becoming completely absent in those channels broken at the carboxy terminus of the S4 helix (e.g. splits 539 and 540) [14]. To investigate such aspect in the channels split at the S2–S3 linker, we used an ‘envelope-of-tails’ voltage protocol in order to analyse their activation rates (Fig. 3). With this procedure, it is possible to evaluate the proportion of channels activated during a depolarization step without the contribution of the overlapping inactivation process [6, 31, 56]. Using the depolarization at 0 mV as a reference, it is observed that the activation time course of the 478 split was clearly accelerated with respect to the wild type, such that equivalent activation rates were attained with the 478 split at − 40 and with the WT at 0 mV. However, the Kv11.1 characteristic initial delay was still maintained in the 478 split channel (inset in the upper panel of Fig. 3b). The acceleration of activation was much more prominent in the case of the 482 split that also exhibited a reduced effect on the activation rates of the changes in depolarization potential (Fig. 3b, c). Furthermore, almost no initial delay is observed with this construct, even at the considerably negative depolarization level of − 60 mV, at which the time course could still be accurately studied because this split combination displays a negatively shifted activation voltage dependence. These results indicate that split channels disconnected at residue 482 in the S2–S3 linker show a reduced ability to reach more distal closed state(s) characteristic of the multi-step sequential process of Kv11.1 activation that cause the initially delayed sigmoidal activation time course [11, 22, 30, 42, 58, 61, 62]. Also, they further emphasize the parallelism between this construct and the split channels sectioned at the base of the S4 helix [14].

**Fig. 4:**
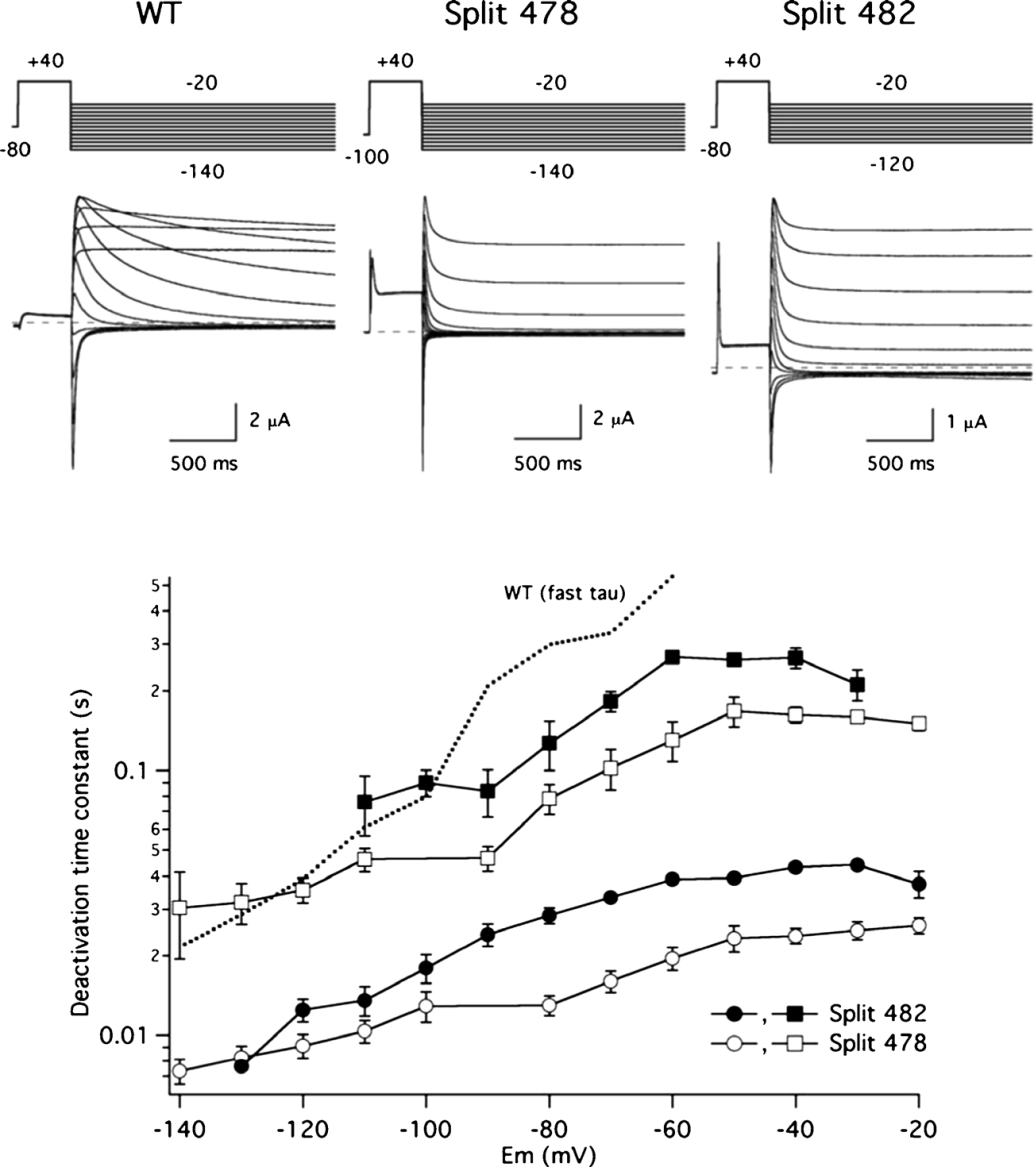
Effect of S2–S3 linker splits on Kv11.1 voltage-dependent deactivation kinetics. Representative families of currents are shown at the top, obtained during steps to potentials ranging from − 20 to − 140 mV in 10-mV intervals, following depolarization pulses at + 40 mV to open (and inactivate) the channels, using the indicated protocol. For clarity, only the first part of the 4-s repolarization steps used to follow the complete decay of the tail currents is shown. The dependence of deactivation rates on repolarization membrane potential is shown at the bottom. Deactivation time constants were quantified by fitting a double exponential to the decaying portion of the tails as described in ‘Materials and methods’. Fast (circles) and slow (squares) deactivation time constants are depicted in the plot. Data corresponding to the fast decaying component from continuous wild-type channels (WT) are shown as a dotted line for comparison

As a further characterization of the S2–S3 linker splits, we also analysed their voltage-dependent deactivation properties using a two-step voltage protocol and fitting the tail currents with two exponential functions (see Methods). As shown in Fig. 4, the deactivation kinetics appeared strongly accelerated and the slopes of the time constant-vs-voltage plots of the splits were smaller than that of the wild-type channel, these effects being particularly marked in the case of the 478 Split. This behaviour has also been observed with single-point mutants in the S4–S5 linker [1, 37, 38, 47], when a break is introduced at the C-terminal section of the linker [14, 32], and when the structural integrity of the so-called N-tail at the beginning of the amino terminus is disrupted [21, 36, 62, 63]. Interestingly, albeit not as clearly as in the case of those splits broken at the S4 helix/S4–S5 linker connection [14], the acceleration of deactivation resulted less marked in the channels split after residue 482, again, emphasizing the existence of some functional similarities between them. It is also interesting to note that almost no effects on the kinetics for the onset of inactivation and on voltage dependence of inactivation were observed when they were studied in the 482 split (Suppl. Fig. 1).

**Fig. 5:**
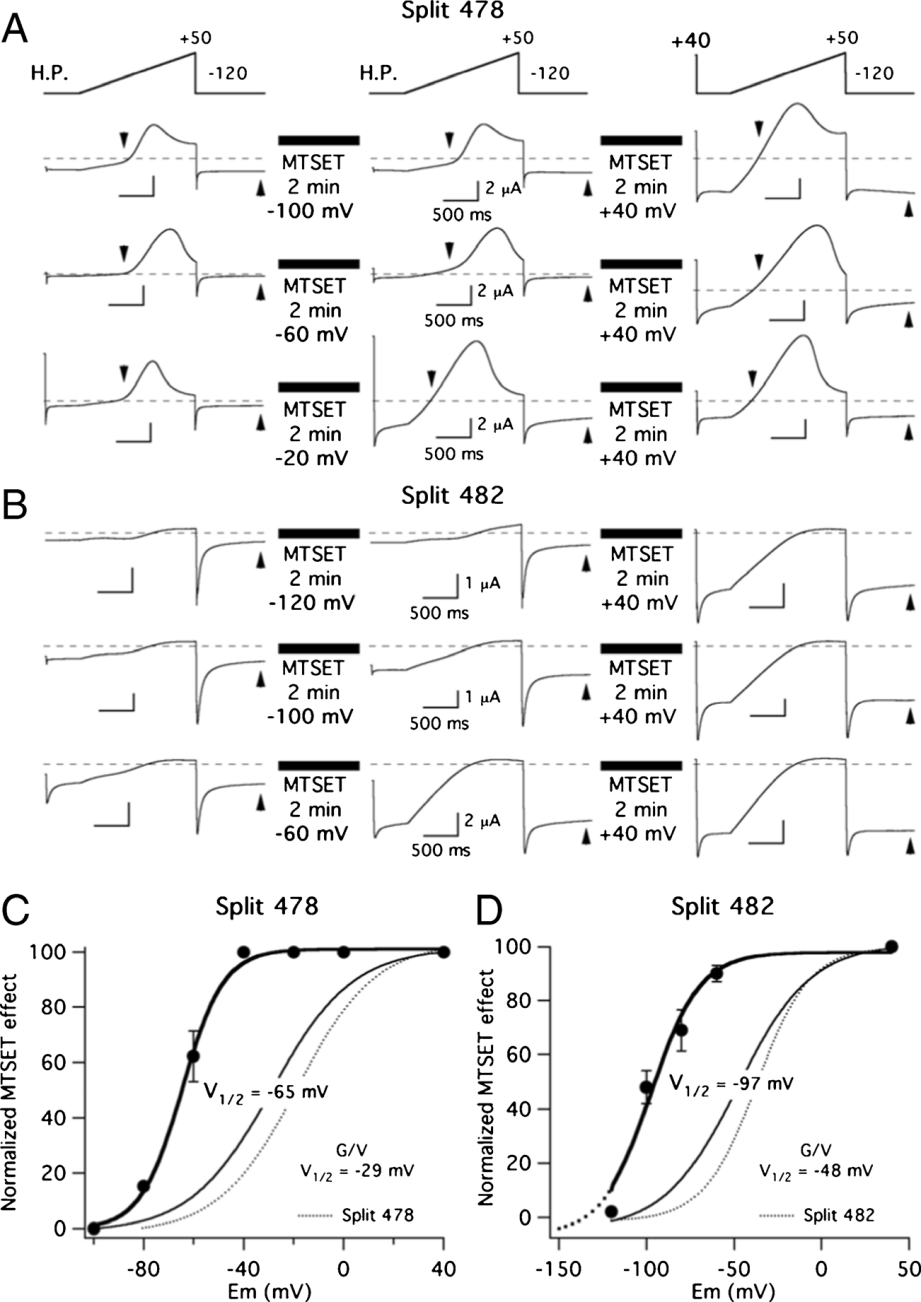
Effect of S2–S3 linker interruptions on voltage dependence of MTSET availability to an engineered cysteine at position 521 in the extracellular part of the voltage sensor S4 helix. **a**, **b** Absence of MTSET effects in cells held at negative potentials not submitted to repetitive depolarization pulsing, and determination of the voltage range at which the MTS reagent induces its effects under steady-state conditions. Current traces obtained in response to the indicated voltage protocols are shown for 478 (**a**) and 482 (**b**) splits. No pulses were applied during the 2-min periods indicated by black boxes at which the cells were continuously held at the indicated potential. A high K^+^ extracellular solution was used to maximize the currents due to the small current magnitudes obtained with the Split 482 construct (see ‘Materials and methods’). Arrowheads are used to indicate the time at which the current variations were estimated quantifying either the magnitude of the final versus peak current during the voltage steps following the ramp pulses or the rectification factor during the ramps as indicated in Methods. Similar results were obtained in both cases. **c**, **d** Voltage dependence of the MTSET effect. Magnitudes of MTSET-induced variations in currents measured (**b**), following a 2-min exposure to 1 mM of MTS reagent without pulsing at the holding potentials indicated in the abscissa were normalized to those observed at a positive potential value of + 40 mV. Due to the irreversibility of the MTSET effects, only one reagent application was performed and a single holding potential value (followed by a positive control at + 40 mV) was checked in each cell studied. Data from three to six cells were averaged for every single point. Some error bars are smaller than the symbols. Continuous lines linking the data points correspond to fits using a Boltzmann function as indicated in ‘Materials and methods’. The corresponding *V*_1/2_ values are shown on the graphs. *G*/*V* plots from the same constructs obtained from fits to tail current data and *V*_1/2_ values derived from them are also shown as indicated. Note the similarity of these plots and those from the same splits without the mutations I521C, C445V and C449V (dotted lines; reproduced from split channels curves in Fig. 2) introduced to study the MTSET-induced effects (see ‘Materials and methods’ section)

To check for the possible impact of the S2–S3 linker interruptions on voltage sensor conformational changes, we studied their effect on voltage-dependence of MTSET accessibility to an engineered Cys residue located in the upper S4 helix of the VSD. For this purpose, we introduced a Cys at position 521 to allow us to follow its modification in the presence of MTSET [14]. The results of the experiments to determine the steady-state voltage dependence of MTSET accessibility to Cys 521 are shown in Fig. 5. In this case, after obtaining control currents using a brief voltage ramp as a stimulatory step, cells were held at different potentials and exposed to MTSET for 2 min without pulsing. Subsequent application of the ramp pulse indicated the extent of channel modification at each potential. As an internal control, in all cases, a second period at a holding potential of + 40 mV was used to quantify in every individual cell the maximum MTSET effect (Fig. 5a, b). It can be observed that no significant effects of MTSET took place when cells expressing the 478 split were held at − 100 mV and when the 482 split-expressing cells were held at − 120 mV, the most negative potential at which a stable recording was achieved with this construct. Furthermore, maximum MTSET-induced effects analogous to those obtained at + 40 mV occurred with holding potentials of − 40/− 20 mV in the 478 split and at around − 60 mV in the 482 split. Plotting the relative magnitude of the MTSET effect against the holding potential indicated that the *V*_1/2_ for MTSET-induced modification amounted to − 65 mV for the 478 split, a value quite similar to the − 58 mV recently measured in control continuous I521C channels [14]. The *V*_1/2_ value was shifted to − 97 mV for split 482 (Fig. 5c, d). In both cases, these values appeared clearly displaced to the left compared to obtained in the *G*/*V* plots of the same splits, indicating that the movement of S4 in the VSD is tracked with the MTS reagent [14, 19, 24, 25, 42, 66]. Furthermore, these data demonstrate that the hyperpolarized *G*/*V* voltage dependence exhibited by the 482 split is accompanied by a shift in the voltage-dependent availability of the I521C residue, also suggesting that the energetic barrier for structural reorganizations and motion of the VSD is lowered by breaking the covalent backbone at this level.

**Fig. 6:**
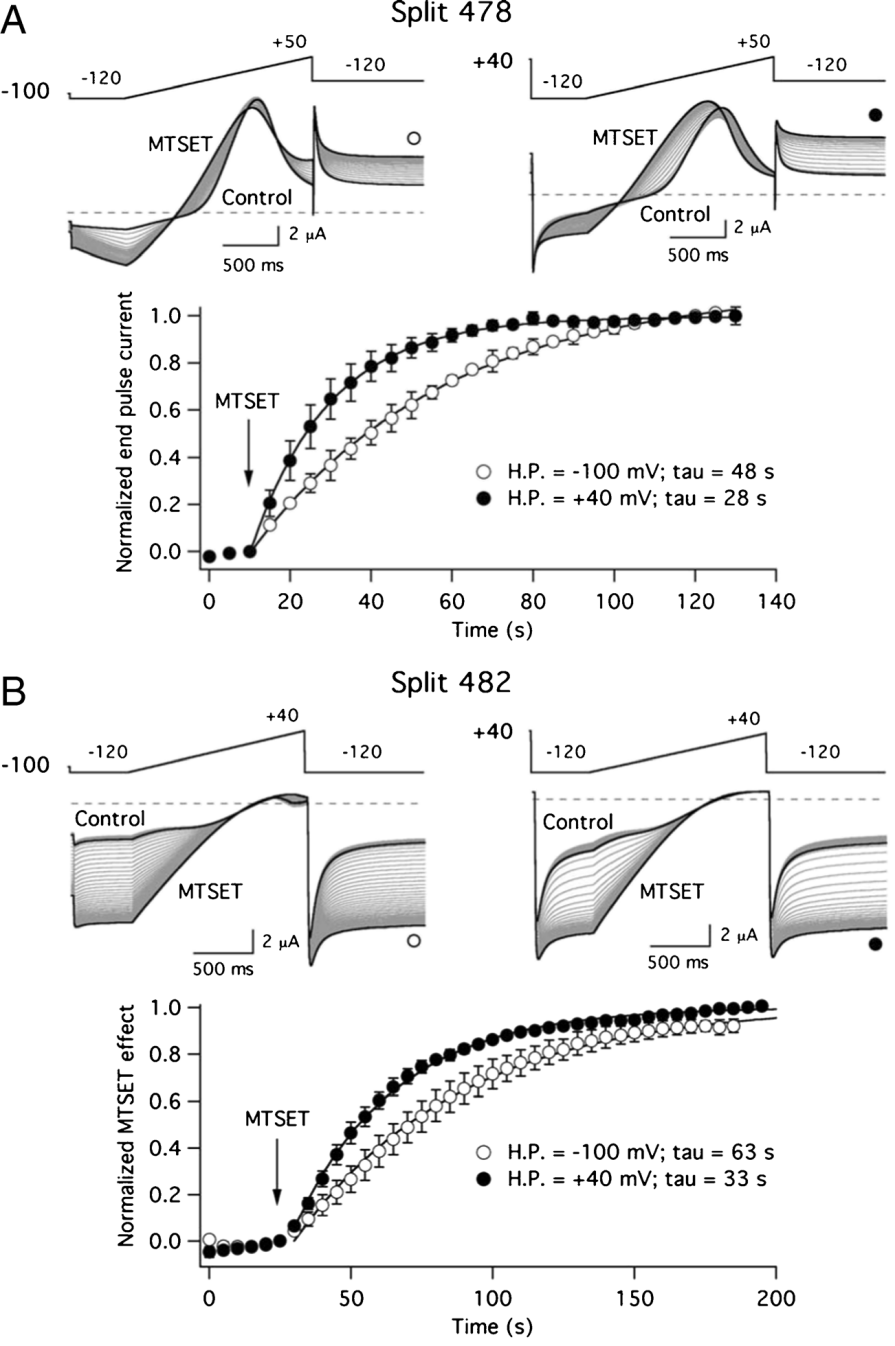
Effect of S2–S3 linker interruptions on MTSET-induced modification rates of engineered cysteine 521. **a** Split 478. Current traces shown at the top were obtained in response to the indicated protocol that was repeated at 5-s intervals. Current traces corresponding to times immediately before application of 1 mM MTSET and at the end of the MTSET exposure using hyperpolarizing (− 100 mV) and depolarizing (+ 40 mV) holding potentials are highlighted as thicker black lines. The bottom plot illustrates the time course of MTSET-induced modifications normalized to those observed at the end of the treatment. The changes in the peak/end current relationship during the − 120 mV voltage step were used to generate the plot. Mono-exponential fits to the data are shown superimposed on the symbols. The values of the corresponding time constants (tau) are indicated in the graphs. **b** Split 482. Plots represent the representative currents and time course of MTSET-induced modifications as detailed (**a**). A high K^+^ extracellular solution was used with the Split 482 construct

As a final characterization of the S2–S3 linker break effects, we also studied the dynamics of the S4 motion in the splits checking the modification rate of the currents in response to repetitive depolarization ramps during exposure to MTSET. Both hyperpolarized (− 100/− 110 mV) and depolarized (+ 40 mV) holding potentials were used at which the channels would remain either closed or open during the interpulse intervals, respectively, and the I521C residue in the upper S4 helix would rest either essentially buried or maximally exposed to the MTS reagent (Fig. 6). Under these conditions, single exponential fits to the normalized current variations at both potentials yielded quite similar MTSET-induced modification time constants for 478 and 482 splits, that in the case of Split 478 amounted to 48 ± 6 s (*n* = 3) and 28 ± 5 s (*n* = 5) at − 100 and + 40 mV, respectively. At hyperpolarized potentials, the corresponding values were 63 ± 8 s (at − 100 mV, *n* = 4) and 58 ± 14 s (at − 110 mV, *n* = 3) for Split 482, that also exhibited a modification time constant of 33 ± 3 s (*n* = 6) at + 40 mV. Interestingly, these modification rates were not significantly different from those measured with continuous I521C channels, that corresponded to 77 ± 9 s (*n* = 4) and 36 ± 6 s (*n* = 5) at − 100 and + 40 mV, respectively. Altogether, these data suggest that when the differences in voltage dependence are taken into account to ensure that the channels remain at a similar level of activation at rest, the breaks in the S2–S3 linker do not drastically alter the ability of the S4 segment to translocate across the membrane.

### Impact of S3–S4 loop interruptions on functional characteristics of the Kv11.1 channels

**Fig. 7:**
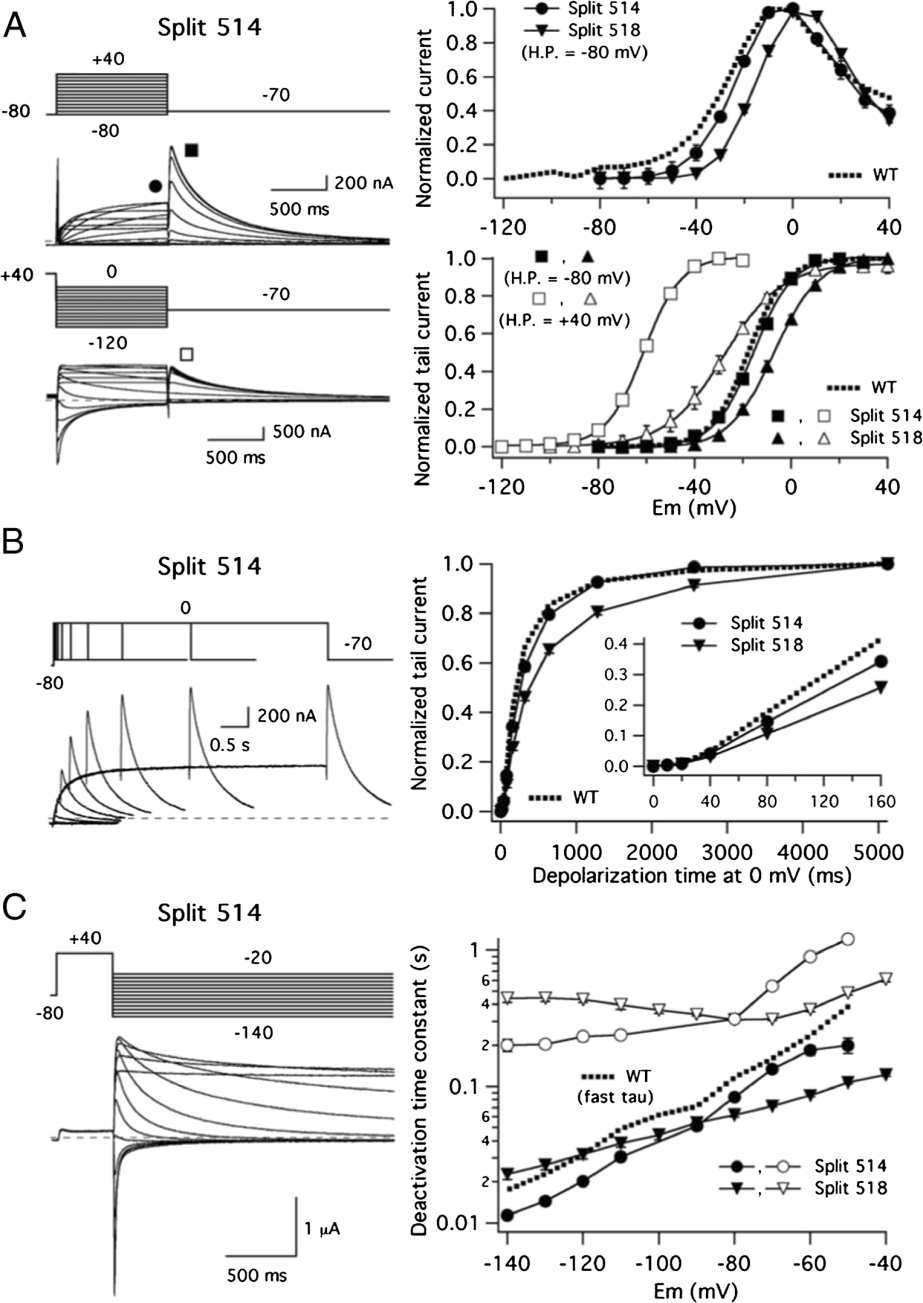
Activation and deactivation gating characteristics of Kv11.1 channels split at the S3–S4 linker. **a** Voltage dependence of activation gating. Representative membrane currents from an oocyte expressing the 514 split channel are shown on the left. Currents were recorded using the protocols shown on top of the traces at − 80 and + 40 mV holding potentials as indicated. Currents are shown without leak subtraction. Averaged *I* vs *V* relationships measured at the end of the depolarization step (signalled by the solid circle on the left) and at the peak of the tail current (*G*/*V* plots, squares on the left) are shown in the right panels. In this case, not only data from split 514 (circles and squares), but also those obtained from split 518 (triangles) are depicted. Data from continuous wild-type channels obtained at a holding potential of − 80 mV are also shown as dotted lines for comparison. **b** Comparison of activation rates at 0 mV. Representative membrane currents from an oocyte expressing the 514 split channel are shown on the left. Averaged plots of normalized tail current magnitudes versus depolarization time at 0 mV are shown on the right both for 514 (circles) and 518 (triangles) splits. Values from continuous wild-type channels are shown as a dotted line for comparison. An expansion of the initial tens of ms to highlight the early current delay in the sigmoidal activation time course is shown in the inset. **c** Voltage-dependent deactivation kinetics. A representative family of currents from an oocyte expressing the 514 split channel is shown on the left. The dependence of deactivation rates on repolarization membrane potential is shown on the right. Fast (closed symbols) and slow (open symbols) deactivation time constants for split 514 (circles) and split 518 (triangles) splits are depicted in the plot. Data corresponding to the fast decaying component from continuous wild-type channels are also shown as a dotted line for comparison

The activation and deactivation gating characteristics of the two functional splits interrupted in the extracellular S3–S4 loop were studied using protocols analogous to those detailed above for the 478 and 482 splits. The data are summarized in Fig. 7. It can be observed that in the case of the 514 split channel, the voltage dependence of activation was almost identical to that of wild-type channels, also including a similar level of mode-shift behaviour (Fig. 7a, see [14]). The analogy between the split and WT kinetics can also be extended to the channel activation rates. Thus, using a depolarization at 0 mV as a reference, the activation time course of the split was superimposable on that of WT, and maintained the characteristic initial delay and the sigmoidal activation time course typical of Kv11.1 (Fig. 7b, see [11, 14, 22, 30, 42, 51, 58, 64]). Finally, similar voltage-dependent deactivation rates (Fig. 7c) and very little variations in inactivation kinetics (Suppl. Fig. 2) were also observed when compared with WT channels. Remarkable similarities in gating were also noticed with the 518 split, although in this case, some subtle differences were detected. Thus, the potential for half-maximal isochronal activation of this split construct appeared slightly shifted to more positive voltages. This was accompanied by a reduction of the strength of the mode-shift behaviour. We also observed a slight slow down of the activation kinetics, although the initial delay of the activation time course remained the same, suggesting that the channels presented a similar ability to reach the more distal closed state(s). Finally, although relatively similar deactivation rates were detected, the slope of the deactivation time constant vs voltage plot was reduced, suggesting that the coupling between the voltage-sensing machinery and the gating process was slightly altered.

**Fig. 8:**
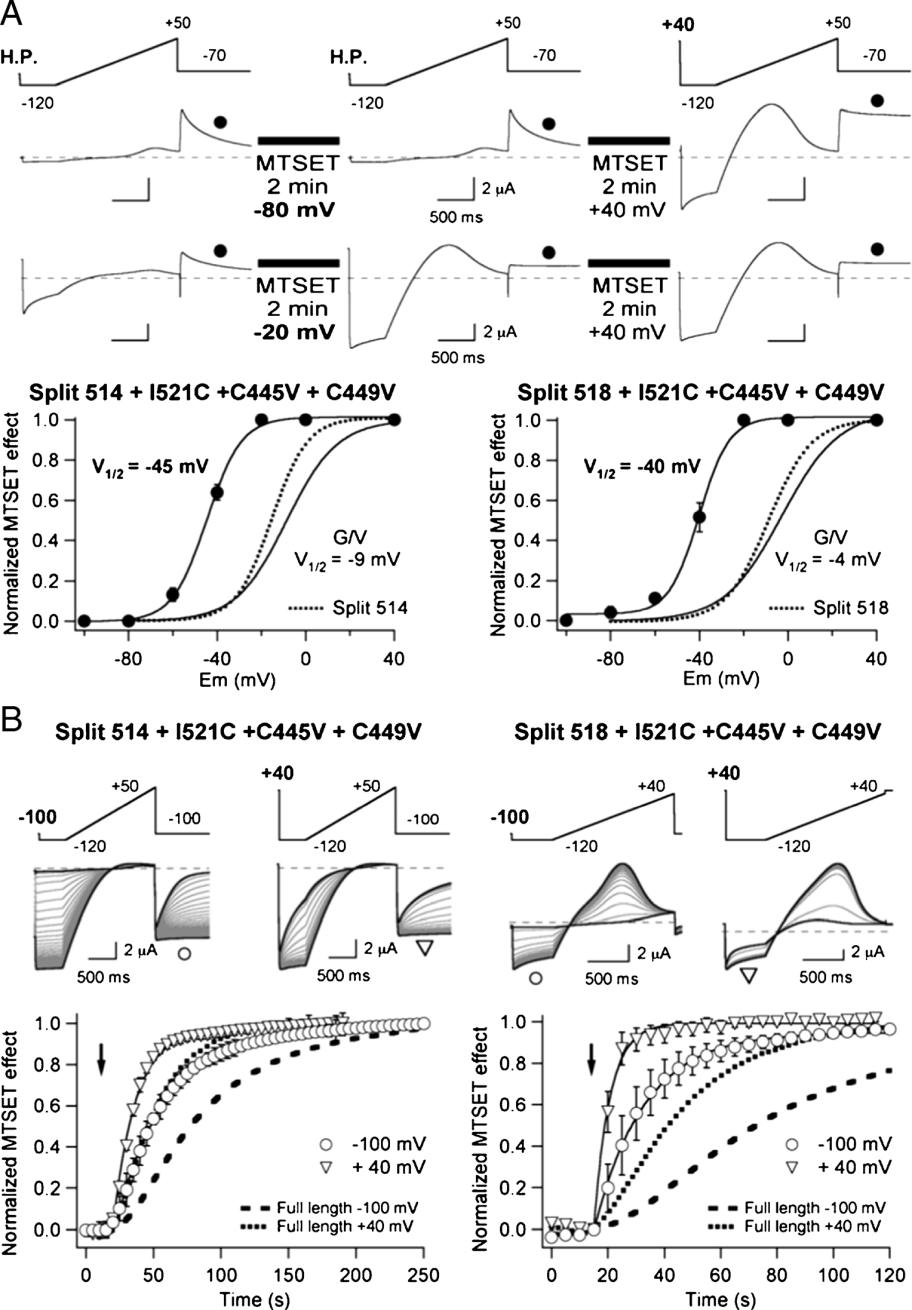
Effect of S3–S4 linker interruptions on MTSET availability to an engineered cysteine at position 521 in the extracellular part of the voltage sensor S4 helix. **a** Steady-state voltage dependence of MTSET effects. Representative current traces from two oocytes expressing 514 split channels held at the indicated potentials and not submitted to repetitive depolarization pulsing are illustrated at the top. No pulses were applied during the 2-min periods indicated by black boxes at which the cells were continuously held at the indicated potential. Measurements of current variations at the end of the repolarizing step (circles) were used to determine the voltage range at which the MTS reagent induces its effects under steady-state conditions as shown at the bottom. Plots obtained from split 514 (bottom left) and split 518 channels (bottom right) are shown. Continuous lines linking the data points correspond to fits using a Boltzmann function as indicated in ‘Materials and methods’. The corresponding *V*_1/2_ values are shown on the graphs. *G*/*V* plots from the same constructs obtained from fits to tail current data and *V*_1/2_ values derived from them are also shown. Note the similarity of these plots and those from the same splits without the mutations I521C, C445V and C449V (dotted lines; reproduced from Fig. 7a). **b**MTSET-induced modification rates of cysteine 521. Current traces shown at the top were obtained in response to the indicated protocols that were repeated at 5-s intervals. Data from 514 (left) and split 518 channels (right) held at − 100 and + 40 mV are shown as indicated. A high K^+^ extracellular solution was used to maximize the currents of the Split 514 construct. Current traces corresponding to times immediately before application of 1 mM MTSET and at the end of the MTSET exposure are highlighted as thicker black lines. Plots of the time course of MTSET-induced modifications normalized to those observed at the end of the treatment are depicted at the bottom. The changes in the peak/end current relationship during the − 120 mV voltage steps were used to generate the plots. The time of MTSET introduction into the recording chamber is marked with an arrow. Mono-exponential fits to the data are shown superimposed to the symbols. Data from I521C full-length continuous channels at − 100 and + 40 mV are shown for a better comparison as dashed and dotted lines, respectively. Note the different time scale of the split 514 and split 518 plots in the abscissa

As a final test of the possible impact of the interruptions in the S3–S4 loop on channel function, we also compared the voltage-dependent MTSET accessibility to the Cys introduced at position 521 of the S4 segment. As shown in Fig. 8a, both in the 514 and 518 splits, I521C remains extracellularly unexposed to MTSET at − 100 mV, but exposed at + 40 mV. Also, the little variations in activation voltage dependence observed in the 518 split were reflected in the *V*_1/2_ value for current modification of this construct as estimated from the steady state MTSET effects vs voltage plots. Remarkably, when the dynamics of S4 motion were studied checking the modification rate of the currents at hyperpolarized and depolarized holding potentials, a significant acceleration of the modification time course was observed in both constructs (Fig. 8b). Thus, the 77 ± 9 and 36 ± 6 s time constants for S4 equilibration measured with continuous wild-type channels at − 100 and + 40 mV, respectively, were lowered to 38 ± 5 s (*n* = 4) and 17 ± 5 s (*n* = 4) in the 514 split. This reduction became much more marked in the case of the 518 split, that showed time constant values of 18 ± 9 s (*n* = 3) and 5.6 ± 3 s (*n* = 3) at − 100 and + 40 mV, respectively. These data indicate that the breaks in the extracellular S3–S4 linker do not greatly modify the ability of the VSD to track the channel gating machinery. However, they also suggest that disruption of the S3–S4 linker continuity favours a more rapid exposure of the upper S4 helix to the external membrane impermeable MTS reagent.

## Discussion

In this work, we present data that extend our recently reported results, demonstrating that not only a combination of the Kv11.1 VSD and PD modules expressed in *Xenopus* oocytes as two independent proteins (S4–S5 linker splits, [14, 32]), but also Kv11.1 split channels broken within the VSD at the level of the S2–S3 and S3–S4 linkers, give rise to fully functional channels. The concept of a modular arrangement of Kv channels as a combination of two functionally autonomous VSD and PD modules appears firmly established [2, 7, 28, 52, 68, 69] and could explain the functionality of the S4–S5 linker splits, considering that it is the S4–S5 linker which bridges the voltage sensing and pore domains. However, providing that the two channel halves remain in the oocyte as independent proteins in a single complex, as demonstrated in the case of the highly homologous Kv10.1 channel [32], it does not easily explain how a functional channel is assembled from two non-covalently joined demi-channels split at other levels outside the S4–S5 linker. In Kv channels, it has been previously shown that a Kv1.2 channel in which the S3–S4 linker has been enzymatically cleaved maintains an almost normal activity [44]. Functional expression in *Xenopus* oocytes of conducting and non-conducting *Shaker* channels split at the S3–S4 linker has been also reported [43], indicating that the structural integrity of this linker is not indispensable for proper synthesis, processing, assembly, quality control and trafficking to the plasma membrane. However, to our knowledge, the successful expression of a S2–S3 linker split Kv channel has not been previously reported. Results of in vitro translation and translocation experiments to understand the membrane topogenesis of the Kv channels have revealed that the membrane insertion of VSD and PD take place independently of each other [49], but also suggest some differences in the S1 to S4 requirements for integration of different Kv VSDs. Thus, the S3 segment of Kv1.3 and KAT1 does not insert into the membrane by itself [48, 57] and has low membrane insertion activity in KvAP [35], but that of *Shaker* efficiently integrates into the membrane [70]. Indeed, in this case, S3 mediates the insertion of the S3–S4 segment in the absence of S2 that acts as an essential factor for optimal integration of this segment in KAT1 [48, 49]. Albeit more complex scenarios are possible, our data with S2–S3 split channels indicate that in Kv11.1 an S2–S3 covalent connection is not necessary for effective insertion of S3, this being consistent with the recognized topogenic properties of segment S2 [48, 49, 57] directing the proper insertion of the demi-N channel half. They would also suggest that either an autonomous S3 or the coordination of S3 and S4 in the S3–S4 segment could also act to insert the demi-C half. Note that even though an intrinsic topogenic activity of S3 exists, the possibility that in the presence of S4 a more efficient membrane integration of S3 takes place [70] may not be excluded. Regarding helix S4, it has been indicated that in Kv1.3 this segment shows a weaker integration ability than other transmembrane segments [57], whereas it efficiently inserts into the membrane in the case of KvAP with the help of its signal anchor type I topogenic function [35]. KAT1 S4 does not integrate into the membrane by itself, but it synergistically inserts with S3 in the case of KAT1 and Kv1.3 [48, 49, 57]. Finally, the membrane unstable S4 of *Shaker* seems to cooperatively act with S3 for fully efficient membrane insertion, and the electrostatic interactions among S2, S3 and S4 play a critical role not only stabilizing the VSD, but also for its optimal membrane integration [70]. Our results with the S3–S4 split suggest that the S4 segment of Kv11.1 does not need the contribution of the initial VSD helices for proper transmembrane insertion. They also demonstrate that in Kv11.1, the presence of S4 and/or any electrostatic pairing with its positive residues is not essential for post-translational membrane integration of S3. If in this case the recognized ability of S4 to insert into the membrane [35] directs the integration of the C-terminal half of the channel, or if it depends on the signal anchor activity of S5 [57], remains to be determined. Further work is necessary to determine the relative relevance of each transmembrane segment for the membrane integration of the whole channel structure in this multiple topogenic system.

The study of the functional properties of S2–S3 linker 478 and 482 splits and the comparison with those of the continuous wild-type channel demonstrate that, particularly in the case of the 482 split, some striking analogies exist with the behaviour of the splits interrupted at the C-terminal end of the VSD S4 transmembrane helix (e.g. 539 and 540 splits [14]). Thus, although a similar half-maximal isochronal activation was found in the 478 split with respect to that of the wild-type channel, a clear shift to more hyperpolarized potentials was exhibited by the 482 split, such as happened with the 539 and 540 splits. A reduction in the amount of equivalent gating charges (*z*_*g*_ values) estimated from the slope of the *G*/*V* curves and an abolishment of the mode shift behaviour was observed in both S2–S3 linker splits. Furthermore, both constructs showed a clear acceleration of the activation time course in response to membrane depolarization that in the 482 split was more marked and accompanied by a complete absence of the initial delay that precedes the exponential phase of activation typical of Kv11.1. All these properties emphasize the parallelism with the altered gating properties recently observed with channels split at the beginning of the S4–S5 linker [14]. Interestingly, whereas split 478 shows a prominent acceleration of deactivation that more closely resembles that observed in the splits interrupted at the C-terminal end of the S4–S5 linker, this acceleration is slightly reduced in the 482 split, a situation also encountered in those constructs interrupted at the S4 helix/S4–S5 linker connection [14]. The shifts in voltage dependence of current activation were accompanied by similar alterations in the voltage dependence MTSET accessibility to a Cys residue introduced in the upper part of the S4 helix, even though the breaks in the S2–S3 linker do not appear to greatly influence the ability of the S4 segment to dynamically translocate across the membrane in response to depolarizing pulses. Altogether, these data suggest that, albeit less markedly, channels split after residue 482 in the S2–S3 linker resemble the uncoupled gating phenotype of those split at the C-terminal end of the VSD S4 segment.

It is interesting to note that the relatively different behaviour of the 482 split with respect to Split 478, indicates a certain specificity of the S2–S3 linker break position triggering alterations of the kinetic properties, also suggesting that the more marked kinetic modifications observed with the 482 split should not be due to an impairment of the construct ability to assemble in the membrane. On the other hand, the possibility that breaking the linker in other positions may cause even more drastic alterations can’t be excluded. The recently reported cryo-EM structures of Kv11.1 and its highly homologous Kv10.1 indicate that a particularly long S2–S3 linker constitutes a conserved feature of the KCNH family [65, 67] and demonstrate that the most amino terminal region of the channel (the N-tail) lies in close contact not only with the S4–S5 and the C-linkers, but also with the S2–S3 linker [14]. However, the exact role of the S2–S3 linker in Kv11.1 gating remains unknown. We have recently proposed that some interactions between the base of the VSD and the PD in which the N-tail acts as a coupling factor, participate in the modulation of Kv11.1 gating and/or in the voltage-dependent electro-allosteric mechanism that transduces VSD reorganizations to the operation of the PD gate [14]. Given the contacts and central positioning of the N-tail with respect to those regions [14, 65] and the relatively similar impact of N-terminal S4–S5 linker and S2–S3 linker breaks demonstrated here, it is tempting to speculate that the dynamic interplay between the N-tail and all these regions, including the S2–S3 linker, constitutes a crucial component of the Kv11.1 gating machinery.

Our data indicate that the S3–S4 linker interruptions have very little impact on the functional behaviour of Kv11.1. The better exposure to MTSET of Cys 521 in the upper S4 helix can be easily explained if the S3–S4 linker splits allow for a less tightened pathway for the movement of the MTS reagent, this also being consistent with a slightly better access of MTSET in the case of the 518 split, in which the break is located only three residues apart from the cysteine modified at position 521. The solved structure of the S3–S4 linker in Kv10.1 demonstrates that it is constituted by a very short, one amino acid long turn [67]. In this context, our data suggest that the short and probably rigid characteristics of the extracellular S3–S4 linker are not an essential factor for proper working of the voltage sensing machinery. Unfortunately, in the only available tridimensional structure of Kv11.1, the structural organization of the S3–S4 linker is not solved [65]. This also opens the possibility that in this case, a highly disordered and basically flexible region is present in the linker that might not be greatly affected by breaks of the covalent backbone. Therefore, until a more precise structural organization of the S3–S4 linker is available, its exact contribution to Kv11.1 function remains an unanswered question.

## Electronic supplementary material

Supplementary Fig. 1 (DOCX 188 kb)

Supplementary Fig. 2 (DOCX 189 kb)
