# Supplementary material for: Functional characterization of Kv11.1 (hERG) potassium channels split in the voltage-sensing domain
Source: Pflugers Arch. 2018 Mar 23;470(7):1069–85. doi: 10.1007/s00424-018-2135-y (PMC6013512; doi:10.1007/s00424-018-2135-y)

**Supplementary Figures**

**Supplementary Fig. 1.** Minimal changes in inactivation kinetics are caused in channels split after residue 482 in the S2-S3 linker. (A) Voltage-dependence of inactivation. A family of current traces recorded in standard in OR-2 medium using the triple pulse protocol illustrated at the top (see Materials and Methods section), is shown on the left. Only currents obtained during the last part of the pulse sequence as highlighted by the grey box are illustrated in the graph. Currents recorded without leak subtraction are shown. Data points corresponding to capacitive transients during the initial three ms following the onset of the voltage steps have been deleted for clarity. Inactivating currents during the final pulse were fitted to a single exponential function and extrapolated to the start of the pulse (dashed lines) to estimate initial current magnitude. Uncorrected (open circles) and corrected for deactivation (closed circles) inactivation voltage-dependence plots are shown on the right (see also Materials and Methods section). Curve superimposed to the corrected data represents a Boltzmann fit. A Boltzmann curve obtained from continuous wild-type channels (WT, dashed line) under identical conditions is also shown for comparison. (B) Rates of inactivation. Onset of inactivation at different voltages was studied in high-K^+^ extracellular solution (see Materials and Methods section) to maximize the currents due to the relative small current magnitudes obtained with the Split 482 construct. A triple pulse protocol was used as shown at the top in which the channels were activated and inactivated with a long depolarizing prepulse, followed by a second short pulse to induce their recovery from inactivation and a final test pulse to different voltages at 10 mV intervals to reinactivate them. Only currents obtained during the last part of the pulse sequence as highlighted by the grey box are illustrated in the graph. A plot of time constants for the onset of inactivation as a function of voltage, obtained from single-exponential fits to the decaying portion of the currents during the test pulse, is shown on the right. Data from continuous wild-type channels obtained in the same conditions are also shown as a dotted line for comparison.


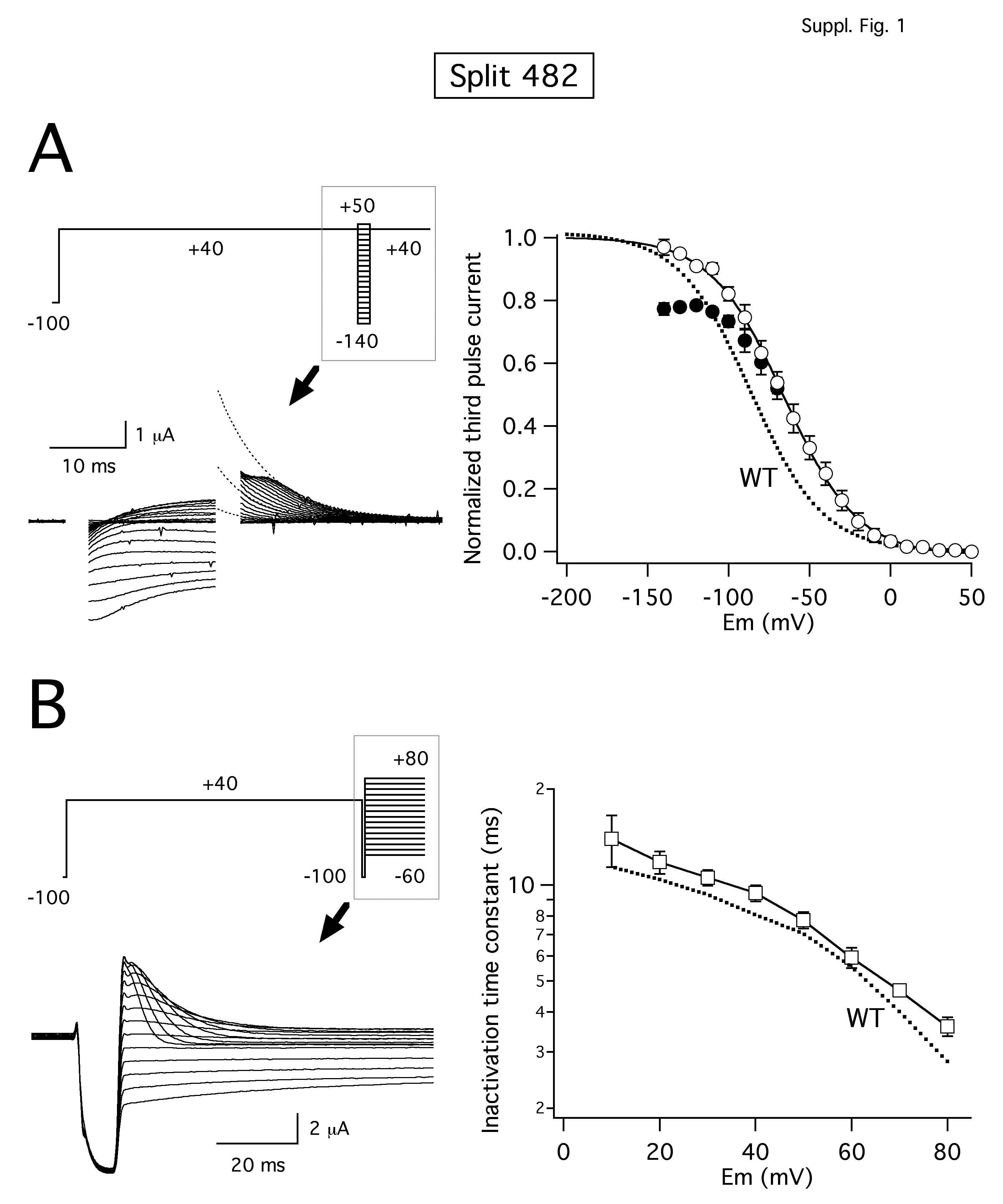

Supplement: Supplementary file 1 — (DOCX 188 kb) [file 424_2018_2135_MOESM1_ESM.docx]
