# Supplementary material for: Functional characterization of Kv11.1 (hERG) potassium channels split in the voltage-sensing domain
Source: Pflugers Arch. 2018 Mar 23;470(7):1069–85. doi: 10.1007/s00424-018-2135-y (PMC6013512; doi:10.1007/s00424-018-2135-y)

**Supplementary Fig. 2.** Characterization of inactivation kinetics of channels split after residue 514 of the S3-S4 linker. (A) Voltage-dependence of inactivation. A family of current traces elicited during the last part of the triple pulse sequence shown at the top as highlighted by the grey box are depicted. Standard OR-2 medium was used for recordings. Uncorrected (open circles) and corrected for deactivation (closed circles) inactivation voltage-dependence plots obtained as detailed in the legend of Suppl. Fig. 1 are shown on the right. A Boltzmann curve obtained from continuous wild-type channels (WT, dashed line) under identical conditions is also shown for comparison. (B) Rates of inactivation. Onset of inactivation at different voltages was studied in standard OR-2 as detailed in the legend of Suppl. Fig. 1. Data from continuous wild-type channels obtained in the same conditions are shown as a dotted line for comparison.


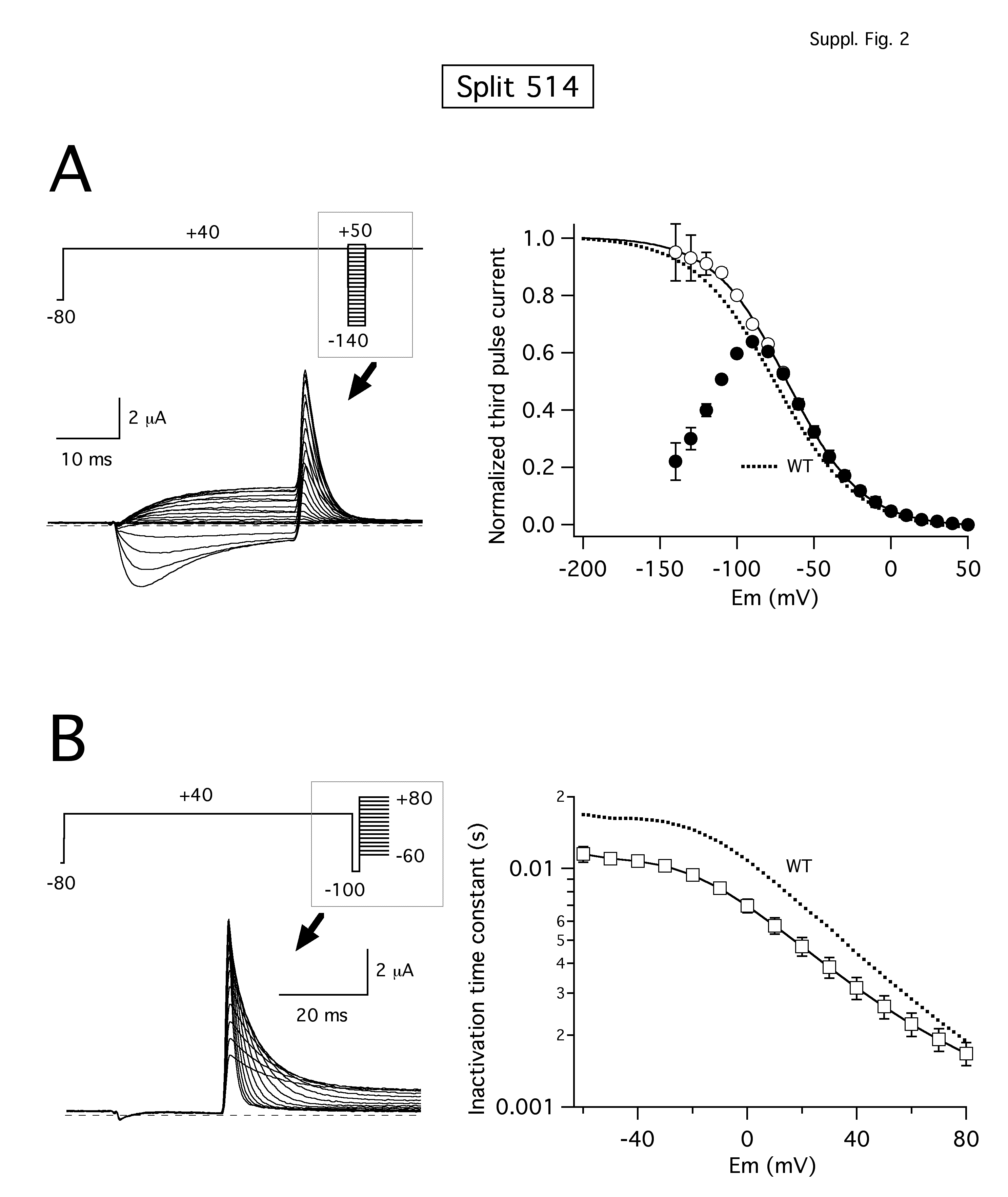

Supplement: Supplementary file 2 — (DOCX 189 kb) [file 424_2018_2135_MOESM2_ESM.docx]
